# Non-inferiority Trial Investigating the Efficacy of Non-steroidal Anti-inflammatory Drugs and Antimicrobial Treatment of Mild to Moderate Clinical Mastitis in Dairy Cows With Long-lasting Udder Diseases

**DOI:** 10.3389/fvets.2021.660804

**Published:** 2021-05-20

**Authors:** Volker Krömker, Anne Schmenger, Doris Klocke, Ellen Maria Mansion-de Vries, Nicole Wente, Yanchao Zhang, Stefanie Leimbach

**Affiliations:** ^1^Department of Veterinary and Animal Sciences, Section for Production, Nutrition and Health, Faculty of Health and Medical Sciences, University of Copenhagen, Frederiksberg, Denmark; ^2^Department of Bioprocess Engineering and Microbiology, Faculty II, Hannover University of Applied Sciences and Arts, Hannover, Germany

**Keywords:** bovine, mastitis treatment, NSAID, chronic, cure, reduction of antibiotic usage

## Abstract

To reduce ineffective antimicrobial usage in the treatment of non-severe clinical mastitis (CM) in cows with long-lasting udder diseases, non-antibiotic therapy with a non-steroidal anti-inflammatory drug (NSAID) was conducted and evaluated in a non-blinded, positively controlled, non-inferiority trial. Therefore, three-time systemic ketoprofen treatment at intervals of 24 h was evaluated in comparison with the reference treatment of solely antibiotic therapy in a field study on nine free-stall dairy farms located in Northern Germany. Cows with previous CM cases in current lactation and/or with long-lasting high somatic cell counts in preceding dairy herd improvement test days were randomly allocated to one of the two treatment groups in cases of mild to moderate CM. Quarter foremilk samples of the affected quarters were taken for cyto-bacteriological investigation before treatment as well as ~14 and 21 d after termination of therapy. Both treatment groups were compared regarding the bacteriological cure (BC) as the primary outcome. Clinical cure (CC) and no CM relapse within 60 d after the end of treatment (no R60) were chosen as secondary outcomes. The study resulted in the following outcomes: *Streptococcus uberis* was most frequently identified in microbiological culture from pre-treatment samples, followed by *Staphylococcus aureus* and *Escherichia coli* and other coliforms. No significant differences between the NSAID treatment and the reference treatment were detected regarding CC and CM recurrence (no R60). Although the sole ketoprofen therapy resulted in a numerically lower likelihood of BC, there were no significant differences to the reference treatment. Considering the selection criteria in this study, the results indicate that in mild to moderate CM cases exclusive treatment with ketoprofen may constitute an alternative to antimicrobial intramammary therapy, providing an opportunity for reduction of antibiotic usage. However, non-inferiority evaluations were inconclusive. Further investigations with a larger sample size are required to confirm the results and to make a distinct statement on non-inferiority.

## Introduction

Due to the increasing development of antibiotic resistance, antimicrobial usage in livestock farming is a critically discussed subject and a matter of public concern. With maximum cure rates in mind, antibiotic overtreatment was propagated for clinical mastitis (CM) over a long period of time (1). It was reported that over 95% of CM was treated with antibiotics in the U.S. (2). Recent available data from Germany suggest that in the field, three out of four CM cases are treated immediately with antibiotics (1). Nevertheless, modern therapeutic strategies also indicate that not every case of mastitis requires antibiotic therapy, and using evidence-based decision criteria, cure rates similar to antibiotic therapy can be achieved with extensive antibiotic savings (3–5).

The goal of antibiotic treatment is to eliminate the causative pathogens from the infected udder quarter and thus achieve a bacteriological cure (BC) (6–9). For chronic disease cases, according to Trevisi et al., antibiotic treatments do not lead to improved animal health and are not appropriate in terms of cost-benefit analysis (10).

The influence of cow-related factors on the BC risk of CM cases treated with antibiotics has been a topic in many studies. It was shown that probability of BC decreases with increasing numbers of previous CM cases in the current lactation (9, 11) as well as high cow somatic cell counts (SCC) prior to CM (9, 11–14). As a result, decreasing the likelihood of BC leads to decreasing efficacy and benefit of antibiotic treatment. Prolonged udder disease is present in cows that have recurrent CM cases or episodes that are interrupted by symptom-free periods with elevated cow SCC - i.e., subclinical mastitis (15, 16). Consideration of the CM history in the current lactation and persistent elevation in cow SCC allows a determination of cows with a low probability of BC following antibiotic treatment. Especially in chronic mastitis cases involving *Staphylococcus (S.) aureus*, BC rates after antibiotic therapy seldom exceed self-cure rates (17).

Rather, it is important to consider whether it is reasonable to define a bacteriological cure as the goal in the treatment of CM. Recent work has shown that 20–30% of all mastitis cases are recurrent mastitis (18, 19). Thus, a large proportion of all CM may be attributed to animals with longer lasting udder infections with clinical flare ups. Infections with *Streptococcus (Sc.) uberis* in particular resolve well, and subsequent infections are largely caused by other strains of the pathogen (19). In conclusion, chronically diseased animals may cure bacteriologically between infections, but a cow with a compromised udder parenchyma will remain chronically ill, showing persistent elevated quarter SCC and will very likely develop clinical signs in turn. Even if a BC can be achieved for some pathogens, it is often not long-lasting. Thus, the value of antibiotics in treatments of such chronic disease cases must be reconsidered.

If possible, those cows should be removed from the herd (20) or treated symptomatically in the case of CM to avoid useless application of antibiotics (21). From a farmer's point of view, such cows, especially the high-yielding animals, are still profitable as long as they show no clinical symptoms and the milk is saleable. Therefore, treatment should focus on decreasing the symptoms of inflammation. In actual practice, however, in the case of chronic mastitis with recurrent clinical flare ups, farmers tend to prolong antibiotic treatment (1, 5).

Non-steroidal anti-inflammatory drugs (NSAIDs) based on ketoprofen are approved in many countries for the adjunctive treatment of clinical mastitis. By preventing the function of the key enzyme cyclooxygenase, ketoprofen inhibits the synthesis of prostaglandin. By NSAID treatment, affected animals benefit from pain relief, which can prevent milk starvation due to insufficient feed intake. Moreover, it has shown positive effects on BC and cows regain physiological milk secretion earlier (22, 23). However, farmers underestimate the positive impact and, against recommendations, tend to omit NSAIDs (5). Demonstrating treatment success with sole NSAID medication in cases of chronically diseased cows could convince skeptical farmers to abandon antibiotic therapy for those animals.

The aim of this study was to evaluate non-inferiority of ketoprofen against antibiotic treatment of mild to moderate CM in cows with long-lasting udder diseases.

## Materials and Methods

### Study Design

This was a randomized non-inferiority study, comparing the outcomes of the test treatment group (ketoprofen treatment) with the outcomes of the reference treatment group (antibiotic treatment). This study is similar to a study we have conducted previously that assessed non-inferiority of an enzyme therapy to AB treatment (24). For better understanding and readability, the study design is described again. The idea of a non-inferiority study is to prove equality of the two treatments by defining an equivalence margin, which specifies a range of values for which the margins between differences in clinical outcome are sufficiently close to be considered equivalent (Δ) (7, 25, 26). The null hypothesis was in our study that a 3-d treatment with ketoprofen is inferior compared to an antibiotic treatment. The alternative hypothesis implied that the 3-d treatment with ketoprofen is non-inferior compared to the antibiotic treatment by more than the equivalence margin of 15% (–Δ) (7, 25):

$$\begin{array}{l} {\text{H}}_{0}:\left[{\text{P}}_{\text{outcome}}\left(\text{ketoprofen}\right)-{\text{P}}_{\text{outcome}}\left(\text{antibiotictreatment}\right)\right]\leq -\text{Δ}\\ {\text{H}}_{\text{A}}:\left[{\text{P}}_{\text{outcome}}\left(\text{ketoprofen}\right)-{\text{P}}_{\text{outcome}}\left(\text{antibiotictreatment}\right)\right]>-\text{Δ} \end{array}$$

Whereby, P_outcome_ is the probability of outcome variables for the ketoprofen and antibiotic treatment. To establish non-inferiority of a test treatment to a reference treatment, the null hypothesis (H_0_) must be rejected in order to have the alternative hypothesis accepted (H_A_). The evaluations of possible study results, applying for this study, were described by Schukken et al. (7).

### Ethical Approval

This study was conducted in accordance with the guidelines on good clinical practice (27). The clinical trial registry number is TVO-2016-V-78. The study complies with the Consolidated Standards of Reporting Trials (CONSORT Checklist).

### Sample Size Determination

Based on former studies of our group and on investigations of Schukken et al. (28), the margin of non-inferiority (Δ) was determined as 0.15 for this study. Furthermore, other scientific working groups previously adopted this value for the non-inferiority margin in CM studies (8, 29). The confidence interval (CI; 95%) approach was used to calculate the required sample size based on the BC rate. In this model, treatments are assumed to achieve similar cure and recurrence rates and we want to assure using the 95% level that the difference is not higher than 15% regarding the margin of non-inferiority (Δ) and the null effect. The sample size was calculated assuming that the antibiotic cure risk was ~50%, and a statistical significance of 5% and power of 80% were chosen. The calculations were performed with the use of StudySize 2.0 (Creostat HB, www.creostat.com) and it resulted in an estimated sample size per group of 137 cases.

Using the estimation of the recurrences due to the higher required sample size, we calculated that if a further 5% of CM cases dropped out of the study post admission, around 145 cases were needed per treatment group. Therefore, a total of 290 cows with CM had to be included.

### Farms and Cows

Inclusion criteria for farms were that farms were motivated to reduce antimicrobials in the treatment of chronic mastitis, participated in the German Dairy Herd Improvement program (DHI), and farm staff were experienced in aseptic sampling in accordance with the guidelines of the German Veterinary Association (30).

The study was conducted on nine free-stall dairy farms located in Northern Germany from October 2014 to September 2018. Herd sizes were between ~160 and 900 lactating Holstein-Friesian dairy cows. The milk production ranged from 9,500 and 11,800 kg/cow/year with bulk milk somatic cell counts between 138,000 and 226,000 cells/ml. None of the farms produced organic milk. All farms used modern milking systems and common hygiene management methods were implemented in daily milking routines (milkers wore gloves, one tissue per cow to clean the teats before milking, teat disinfection after milking). All herds were milked twice a day. A rotary milking parlor was installed on two farms, whereas seven farms owned a herringbone/side by side parlor. All farms fed their cows total mixed rations.

Only cows that met the criteria for chronic, longer lasting udder disease were included in the trial. Every cow had to be registered with a unique ear tag to clearly identify every animal, as stipulated in Germany. Definition criteria were fulfilled in the case of at least three consecutively high cow SCC (> 400,000 somatic cells/ml) in the previous three monthly DHI samplings and/or at least two CM cases in the current lactation. Cows included in the study had shown a period of normal milk secretion before CM occurred. Lactating Holstein-Friesian dairy cows of all parities with CM signs in one or more quarters were eligible for inclusion. Mastitis severity score was defined according to the International Dairy Federation guidelines (31). A CM case was classified as mild (grade 1) if there was only change in the appearance of milk (color, viscosity, consistency; i.e., flaky sediments, watery appearance, discoloration). A moderate CM (grade 2) additionally showed local clinical signs of inflammation of the udder parenchyma (i.e., swelling, heat, pain, redness). In the case of general clinical signs (fever, lack of appetite) the CM was defined as a severe mastitis (grade 3). Only cases of mild to moderate CM were included in the study and only cows free of significant udder, teat, or teat orifice lesions or another additional disease at the same time were used.

### Treatment and Randomization

If a case of CM occurred in an animal that met the definition of a chronically udder-diseased cow, classification of the severity score and the treatment was performed by instructed farm staff. Two different treatment regimens were investigated in the study: animals of the first group, the AB group, received local antimicrobial treatment according to the label of the respective products used on the farms (β-lactam antibiotics); animals of the second group, the NSAID group, received systemic treatment with ketoprofen (three applications at 24 h intervals with 3 mg of ketoprofen per kg bodyweight Kelaprofen® (Veyx-Pharma GmbH, Schwarzenborn, Germany). Cows were randomly allocated to one treatment group based on the last number of their respective barn number (even/odd) and therapy applied following strict asepsis by trained farm personnel. Cows with CM in more than one quarter were also included in the study and all affected quarters received the same therapy. Animals from both treatment groups were not separated for the trial, but were kept under the same conditions on the farms.

### Study Procedure

Farms received a monthly list containing eligible cows based on the monthly DHI results and the farm records of cow CM history of the current lactation. Farm staff were instructed to record clinical data and to fill in treatment protocols in accordance with the study procedure. A cow with a mild or moderate CM case in one or more quarters was identified by the milking personnel and checked for inclusion criteria using the list of eligible cows. If a cow was included in the study, a milk sample was taken in accordance with the guidelines of aseptic milk sampling (30). Following the aforementioned randomization, the animals received the appropriate treatment depending on the assigned treatment group. Each cow was included in the study with only one CM case. Treatment was performed according to the label of the respective product. At day 5 after the end of treatment of a case, the clinical score of the affected quarter was assessed by the milkers. In the case of a deterioration of the clinical appearance, the case was recorded as treatment failure and farmers treated their cow additionally. CM cases without clinical symptoms on day 5 were assessed as clinically cured. These cured quarters were observed from days 6 to 60 after the end of treatment for recurrent CM cases and a quarter foremilk sample was taken in case of return of clinical signs. After treatment, pre-milk samples were collected from all clinically cured quarters on day 14 (±2) and day 21 (±2) after the end of treatment by a veterinarian of the study personnel. All samples were refrigerated and were picked up weekly during farm visits by a veterinarian of our working group. During these regular farm visits, we exchanged information with the herd personnel to resolve inaccuracies and ensure data quality. Any deviations from the study protocol were noted and investigated for eligibility to include in the study. Commonly used cow-level data including lactation number, affected quarter location, cow SCC of the three most recent DHI recordings prior to CM, days in milk (DIM) at CM occurrence, and concurrent diseases and treatments for a period of 30 d after enrolment were recorded.

### Blinding

It was not possible to blind either the study personnel or the farmers/herdspersons to product administration by virtue of the differences in treatment regimens. The laboratory personnel performing cyto-microbiological diagnostic examinations were unaware of the treatment given to the quarters being sampled. But due to the study design it was not possible to blind either the farm staff or the farmer as these people implemented the treatment depending on the treatment group.

### Laboratory Procedure

All milk samples were collected aseptically and were stored below 8°C until analysis. Ly20, containing boric acid as the preserving agent, was used in test tubes (30). The samples were sent to the microbiological laboratory at the University of Applied Sciences and Arts Hannover (Germany). Microbiological examinations were performed in accordance with the guidelines of the German Veterinary Association (30), which are similar to National Mastitis Council recommendations (31). From each milk sample, 10 μl was plated onto one quadrant of an esculin blood agar plate (Oxoid, Germany) and incubated for at least 48 h at 37°C under aerobic conditions. By the assessment of Gram staining, morphology of the colonies and cells, hemolysis patterns, esculin hydrolysis, and activity of catalase (3% H_2_O_2_; Merck, Germany), an initial evaluation of the grown colonies was performed. Subsequently several biochemical tests were done to determine the growing microorganisms. The clumping factor test (DiaMondiaL Staph Plus Kit, Sekisui Virotech, Germany) instead of the coagulase test was used to differentiate presumptive *Staphylococcus* (*S*.) *aureus* from non-*aureus* staphylococci (N*a*S). Different esculin-negative streptococci were distinguished by the serological tests for Lancefield Group B [*Streptococcus (Sc.) agalactiae*], C (*Sc. dysgalactiae*), and G (DiaMondiaL Streptococcal Extraction Kit Sekisui Virotech, Germany). To differentiate between *Sc*. *uberis* and *Enterococcus* spp. the modified Rambach agar according to Watts et al. (32) was used. Gram-positive, beta-hemolytic, catalase-negative irregular rods with a V- or Y-shaped configuration were identified as *Trueperella (T.) pyogenes*. Coryneform bacteria form small colonies on esculin blood agar. They are Gram-positive and catalase-positive. Both, *T. pyogenes* and coryneform bacteria are asporogenic. *Bacillus* spp. form large colonies on esculin blood agar. *Bacillus* spp. are Gram-positive, catalase-positive rods and can form endospores. Coliform bacteria are Gram-negative, catalase-negative, and cytochrome oxidase-negative (Bactident oxidase, Merck, Germany) rod-shaped bacteria, which can metabolize glucose fermentatively (OF basal medium with the addition of D (+)-glucose monohydrate, Merck, Germany). On Chromocult Coliform Agar (Merck, Germany), *Escherichia* (*E*.) *coli* forms blue colonies under aerobic incubation at 37°C for 24 h, other coliforms form pink-red colonies. *Klebsiella* spp. are immobile during the performance of the OF test. Pseudomonads were identified as Gram-negative, catalase-positive, cytochrome oxidase-positive rod-shaped bacteria that break down glucose oxidatively. Yeasts, moulds, and *Prototheca* spp. were differentiated microscopically after subculturing on YGC agar (Merck, Germany). Environment-associated, mastitis-causing microorganisms (*Sc. uberis, E. coli*, N*a*S, *Klebsiella* spp., coliform bacteria, yeasts, *Pseudomonas* spp., and *Prototheca* spp.) were recorded as a microbiologically positive result if ≥5 cfu/0.01 ml were cultured to reduce bias due to contamination. If two pathogens were cultured, the case was included in the study and both microorganisms were documented. A milk sample was considered as contaminated when more than two pathogens were identified, except in cases where also *S. aureus, Sc. agalactiae, Sc. dysgalactiae*, and *T. pyogenes* were cultured. Then only the growth of these pathogens was recorded and the cases were classified as contaminated if the samples contained more than two of these pathogens. Somascope Smart (Delta Instruments, The Netherlands) was used to determine the SCC by flow cytometry.

### Outcome Variables

Primary outcome was BC and secondary outcomes were CC and no CM recurrence within 60 d after the end of treatment (no R60). Quarter somatic cell count was additionally determined to identify quarters with cytological cure (CYC). CC was defined as absence of clinical symptoms in milk, this meant without flaky sediments, watery appearance, or discoloration and on udder quarter, this meant without swelling, heat, redness, or pain at day 5. CM cases of cows, which received additional or different treatment due to deterioration of clinical symptoms within the 5 days or after the end of initial therapy or were removed from the herd due to udder disease were assessed as failure of CC.

Quarters with clinically cured cases were observed for the time frame of days 6–60 after the end of treatment and defined as recurrent quarters when one or more CM cases were detected. A quarter showed no R60 if it was free of CM within the observed time frame.

BC was defined as the absence of the pathogen cultured pre-treatment in both post-treatment samples at days 14 and 21. If a bacterial species other than the pathogen cultured pre-treatment was isolated in the post-treatment samples, the case was still defined as bacteriologically cured. If one post-treatment sample was contaminated, the outcome of the other post-treatment sample was used to determine the BC. If two pathogens were isolated in the pre-treatment sample the case was enrolled as mixed infection and applied as bacteriologically cured if neither of the two pathogens were cultured in both of the post-treatment samples. When a clinically cured quarter suffered from a CM recurrence within days 6–21 after the end of treatment, available post-treatment samples and the recurrence sample were used to determine BC.

CYC was defined as a quarter SCC with <200,000 cells/ml in both post-treatment samples at days 14 and 21. If one post-treatment sample was missing, the CYC of the other post-treatment sample was used to determine the outcome. When a clinically cured quarter suffered from a CM recurrence within days 6–21 after the end of treatment, the CM case was assessed as failure of CYC. Quarters with CM cases experiencing no CC were also included in the analysis as failure of BC and CYC to take the principle of “intention-to-treat” into account (8, 26).

### Statistical Analysis

The data were collected and analyzed using Excel, Office 2010 (Microsoft Corporation) and SPSS (IBM SPSS 26.0.0.0, Armonk, USA). The statistical unit was the CM case of an udder quarter. For every CM case, CC or no CC, R60 or no R60, BC or no BC, and CYC or no CYC (encoded as 1 or 0, respectively) were determined according to the aforementioned definitions, constituting the binary dichotomous-dependent variables. Outcomes were analyzed using generalized linear mixed models including lactation number, DIM, and pathogen (grouped) cultured pre-treatment as important covariates. As clustering was present in the design (i.e., gland within cow, and cow within herd) the analysis was corrected using random effects, but had no relevant influence. The treatment group was the main variable of interest. Statistical significance was assumed at α = 0.05.

The linear predictor was calculated as

$$\begin{array}{r} \text{Logit}\left(\text{outcome}\right)=\text{intercept}+\text{treatment}+\text{lactationnumber}\\ \;+\text{DIM}+\text{pathogen}+\text{her}{\text{d}}^{*}\text{co}{\text{w}}^{*}\text{gland }\left(\text{random}\right). \end{array}$$

BC, CC, no R60, or CYC are the outcomes and lactation number is the lactation number of the included cow grouped as 1, 2, and over 2. DIM is days in milk of the cow at CM occurrence grouped as 0–100, 101–200, and over 200. Pathogens cultured pre-treatment were grouped into *Enterobacteriaceae*, streptococci, staphylococci, other pathogens, contaminated samples, mixed infections, and no growth.

For BC, CC, and no R60, the model was used to calculate least square means of the various treatment groups. Thereby, the differences between treatments were estimated. Confidence intervals of the therapy differences were calculated utilizing the least square means and standard deviation (8).

## Results

### Descriptive Results

A total of 296 CM cases were enrolled in the study. In 17 CM cases, the dataset was incomplete because not all samples were taken (forgotten by the milker) and/or examined (leaked during transport). Antibiotic treatment was applied in 144 CM cases (AB group), whereas 135 cases received ketoprofen (NSAID group) (Table 1). No further treatment had to be initiated in any case due to worsening of the mastitis severity score. No adverse events of treatment were observed. The median of lactation number for all CM cases amounted to 3 (minimum 1; maximum 11) and of milk yield last DHI before CM occurrence, 31.5 kg (minimum 9.6 kg; maximum 58.0 kg). In 135 CM cases the front quarters and in 144 cases the rear quarters suffered from CM. In 178 cases, mastitis severity was classified as mild and as moderate in 101 cases. A proportion of 15.8% of the CM cases occurred in cows in their first 100 DIM, 43.3% in 101–200 DIM, and 40.9% in over 200 DIM, respectively.

**Table 1:** Number of cows per herd assigned to either the reference group with solely antibiotic treatment (AB) or the test treatment group with solely systemic ketoprofen treatment (NSAID).

**Participation (from–until)**	**Farm**	**Cows per herd (size)**	**AB**	**NSAID**
Oct 2014–Sep 2018	A	270	21	15
Oct 2015–Mar 2017	B	160	6	1
Sep 2017–Mar 2018	C	740	4	5
Oct 2014–Sep 2018	D	180	9	11
Oct 2014–Sep 2016	E	850	24	20
Oct 2014–Mar 2017	F	900	26	43
Oct 2016–Sep 2017	G	250	4	2
Oct 2014–Sep 2017	H	780	28	22
Oct 2014–Sep 2018	I	550	22	16
**Total**	**9**	**4,680**	**144**	**135**

The results of bacteriological culture are presented in Table 2. The pathogen most cultured from the pre-treatment sample was *Sc. uberis* (16.8%), followed by *S. aureus* (15.4%), and coliforms (11.1%). No microbiological growth was found in 62 cases (22.2%), 15 quarters showed mixed infections (5.4%), and 28 samples were contaminated (10.0%). In 53.3% of the mixed infections, *Sc. uberis* was one of the cultured pathogens and in 33.3% N*a*S was one of the isolated microorganisms.

**Table 2:** Bacteriological culture results (*n*= 79 CM) of pre-treatment samples of the reference group with solely antibiotic treatment (AB) and the test treatment group with solely systemic ketoprofen treatment (NSAID).

**Microorganism**	**AB (*n* = 144)**	**NSAID (*n* = 135)**
***Enterobacteriaceae***	**13**	**18**
Coliforms (other than *E. coli* and *Klebsiella* spp.)	9	5
*E. coli*	4	13
**Streptococci**	**32**	**30**
*Sc. Uberis*	23	24
*Sc. Dysgalactiae*	5	3
Other streptococci	4	3
**Staphylococci**	**18**	**30**
*S. aureus*	14	29
N*a*S	4	1
**Other pathogens**	**22**	**11**
Coryneforms	5	3
*Pseudomonas* spp.	5	2
*Prototheca* spp.	4	2
Enterococci	3	1
*T. pyogenes*	2	3
Yeasts	3	0
No growth	31	31
Mixed infections	10	5
Contaminated	18	10
**Total**	**144**	**135**

The treatment groups were similar in terms of the lactation number, DIM, mastitis score, and pathogen distribution (*P* > 0.05). For good measure, herd as random effect, DIM, lactation number, and pathogen cultured pre-treatment were included in the generalized linear mixed models to take these factors into account.

### Bacteriological Cure

Bacteriological cure was determined for 189 CM cases. The remaining cases were excluded because no microorganisms were cultured (62 cases) or pre-treatment samples were contaminated (28 cases). The overall BC rate was 44.4% (84/189). The probability of BC in the AB group was 48.4% (46/95) and in the NSAID group 40.4% (38/94).

**Figure 1:**
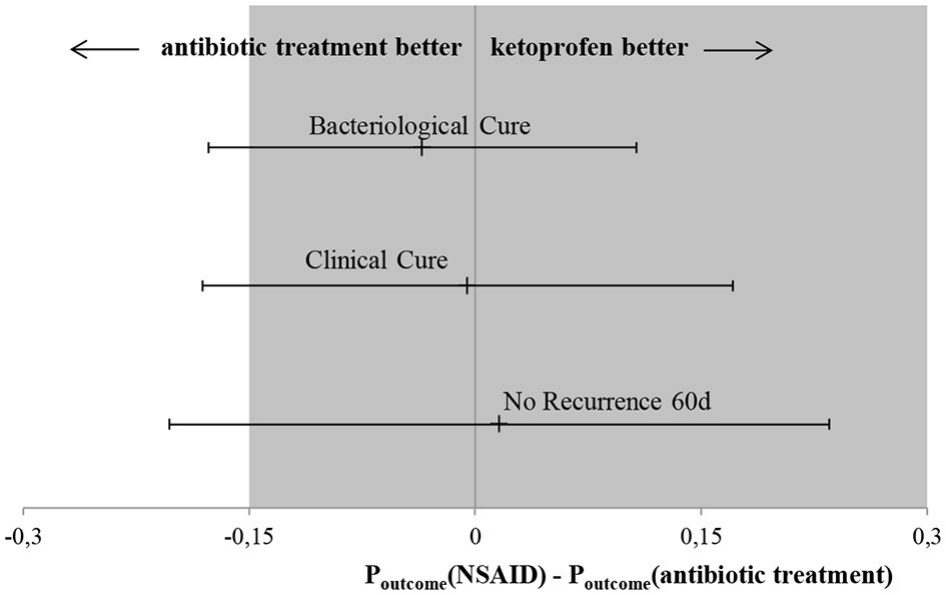
Main results of this non-inferiority trial. Black point presents point-estimate of difference in outcome variables between the test treatments (NSAID) and the reference treatment (AB) with the associated 95% CI indicated by the arrowheads. Dark field represents area of non-inferiority. Bacteriological cure: difference in BC between test treatment (NSAID) in comparison to the reference treatment (AB), the CI spans both 0 and –Δ, non-inferiority is inconclusive and there are no significant differences between the two treatments. Clinical cure: difference in CC between test treatment (NSAID) in comparison to reference treatment (AB), the CI spans both 0 and non-inferiority margin (–Δ), non-inferiority is inconclusive and there are no significant differences between the two treatments. No recurrence 60 d: difference in no R60 between test treatment (NSAID) in comparison to reference treatment (AB), the CI spans both 0 and –Δ, non-inferiority is inconclusive and there are no significant differences between the two treatments.

Results of the generalized linear mixed model showed the least square means of 48.1% for the AB group and 45.6% for the NSAID group. The model demonstrated that no significant differences in BC of the reference treatment AB to the test treatment NSAID were found (*P* = 0.769) (Table 3). Animals with CM within 1 to 100 DIM showed a significantly higher probability of BC than cows suffering from CM > 100 DIM (*P* = 0.028). Cows with staphylococcal infections had a significantly lower BC rate than animals with other pathogens (*P* = 0.028). The point estimate of the calculated differences in BC from the logistic regression and the associated 95% CI is shown in Figure 1. Non-inferiority was inconclusive but very close to non-inferior for NSAID treatment in comparison to the solely antibiotic treatment.

**Table 3:** Final mixed logistic regression model results for the outcome variable bacteriological cure.

**Variable**	**Coefficient**		**OR**	**95% CI**	***P*-value^a^**
	**X**	**SE**			
Intercept	0.616	0.453	1.852	0.758–4.523	0.175
**Treatment**					
AB	0.099	0.336	1.104	0.568–2.143	0.769
NSAID (reference)	0				
**Lactation number of the cow at the day of clinical mastitis occurrence**					
1	1.228	0.554	3.414	1.145–10.181	**0.028**
2	0.170	0.372	1.185	0.569–2.468	0.648
>2 (reference)	0				
**Days in milk at the day of clinical mastitis occurrence**					
0–100	−1.319	0.497	0.267	0.100–0.713	**0.009**
101–200	−0.151	0.373	0.860	0.412–1.796	0.686
>200 (reference)	0				
**Pathogen cultured from the pre-treatment milk sample**					
Mix	−0.818	0.676	0.441	0.116–1.676	0.228
Other	−0.149	0.548	0.862	0.292–2.542	0.786
Staphylococci	−2.222	0.555	0.108	0.036–0.324	**0.000**
Streptococci	−0.656	0.477	0.519	0.202–1.331	0.171
*Enterobacteriaceae* (reference)	0				

a *Significance set at P < 0.05. Bold value indicates significant value*.

*Two different treatment regimens were investigated: NSAID, solely ketoprofen comprising three treatments at an interval of 24 h; AB, antibiotic treatment as usual on the farm according to the label of the respective product*.

### Clinical Cure

The overall CC rate was 50.5% (141/279). The probability of CC in the AB group was 51.4% (74/144) and in the NSAID group 49.6% (67/135).

Results of the generalized linear mixed model showed least square means of 57.9% for the AB group and 57.4% for the NSAID group. Again, no significant differences in CC of the reference treatment AB to the test treatment NSAID (*P* = 0.57) were present (Table 4). Cows suffering from CM with streptococcal infections showed a significantly lower probability of CC than cows affected with other pathogens (*P* = 0.004). The point estimate of the calculated differences in CC from the logistic regression and the associated 95% CI is shown in Figure 1. Non-inferiority was inconclusive but very close to non-inferior in comparison to the reference treatment.

**Table 4:** Mixed logistic regression model results for the outcome variable clinical cure.

**Variable**	**Coefficient**		**OR**	**95% CI**	***P*-value^a^**
	**X**	**SE**			
Intercept	−0.261	0.466	1.298	0.519–3.246	0.576
**Treatment**					
AB	0.021	0.278	1.021	0.590–1.766	0.569
NSAID (reference)	0				
**Lactation number of the cow at the day of clinical mastitis occurrence**					
1	0.166	0.402	1.181	0.535–2.604	0.957
2	−0.286	0.319	0.751	0.400–1.409	0.371
>2 (reference)	0				
**Days in milk at the day of clinical mastitis occurrence**					
0–100	0.348	0.395	1.417	0.651–3.082	0.378
101–200	0.547	0.310	1.728	0.939–3.182	0.079
>200 (reference)	0				
**Pathogen cultured from the pre-treatment milk sample**					
Mix	−0.161	0.637	0.851	0.243–2.984	0.800
Other	−0.015	0.475	0.985	0.387–2.510	0.975
Staphylococci	−0.549	0.437	0.578	0.244–1.366	0.211
Streptococci	−1.145	0.398	0.318	0.146–0.697	**0.004**
*Enterobacteriaceae*	0.176	0.500	1.192	0.445–3.191	0.725
Contaminated	0.148	0.521	1.160	0.416–3.233	0.776
No growth (reference)	0				

a *Significance set at P < 0.05. Bold value indicates significant value*.

*Two different treatment regimens were investigated: NSAID, solely ketoprofen comprising three treatments at an interval of 24 h; AB, local antibiotic treatment as usual on the farm according to the label of the respective product*.

### No Recurrence After 60 d

Only CM cases of cows that reached a CC and were still in milk 60 d after the end of treatment were included in this analysis (11). Of the 141 clinically cured quarters, only two cases were excluded because the cows had been sold within the considered timeframe. Consequently, 139 CM cases were included in the analysis. The overall no R60 rate was 54.7% (76/139). The probability of achieving no CM recurrence 60 d after the end of treatment in the AB group was 58.3% (42/72) and in the NSAID group 50.7% (34/67).

Results of the generalized linear mixed model showed numerically different least square means of 63.3% for the AB group and 64.9% for the NSAID group. However, no significant differences in no R60 of the reference treatment AB to the test treatment NSAID (*P* = 0.556) were found (Table 5). Cows in their second lactation (*P* = 0.009) showed a significantly higher probability of no R60 than cows in the third or higher lactation (*P* = 0.030). Furthermore, animals with CM at the beginning of lactation (<100 DIM) had a significantly lower likelihood of no R60 compared to cows suffering from mastitis later in lactation (*P* = 0.014). CM cases caused by staphylococci and streptococci showed a significantly lower probability of no R60 than CM cases where another or no pathogen was isolated (*P* = 0.022; *P* = 0.038 resp.). The point estimate of the calculated differences in no R60 from the logistic regression and the associated 95% CI is shown in Figure 1. Non-inferiority was inconclusive for the NSAID treatment in comparison to the AB treatment.

**Table 5:** Final mixed logistic regression model results for the outcome variable no recurrence 60 d.

**Variable**	**Coefficient**		**OR**	**95% CI**	***P*-value^a^**
	**X**	**SE**			
Intercept	1.759	1.323	2.461	0.721–8.399	0.149
**Treatment**					
AB	−0.475	0.804	0.932	0.372–2.338	0.881
NSAID (reference)	0				
**Lactation number of the cow at the day of clinical mastitis occurrence**					
1	1.843	0.809	2.939	0.883–9.790	0.079
2	2.137	0.791	3.346	1.126–9.947	**0.030**
>2 (reference)	0				
**Days in milk at the day of clinical mastitis occurrence**					
0–100	−2.815	1.050	0.207	0.059–0.721	**0.014**
101–200	−1.939	0.941	0.734	0.291–1.854	0.510
>200 (reference)	0				
**Pathogen cultured from the pre-treatment milk sample**					
Mix	−1.002	1.744	0.273	0.041–1.838	0.180
Other	0.628	0.980	4.628	0.873–24.542	0.072
Staphylococci	−2.028	1.008	0.184	0.044–0.780	**0.022**
Streptococci	−0.510	0.884	0.246	0.065–0.926	**0.038**
*Enterobacteriaceae*	−1.888	1.767	1.410	0.387–5.135	0.600
Contaminated			0.649	0.179–2.344	0.506
No growth (reference)	0				

a *Significance set at P < 0.05. Bold value indicates significant value*.

*Two different treatment regimens were investigated: NSAID, solely ketoprofen comprising three treatments at an interval of 24 h; AB, antibiotic treatment as usual on the farm according to the label of the respective product*.

### Cytological Cure

The overall CYC was 3.9% (11/279). The probability of CYC in the AB group was 4.2% (6/144) and in the NSAID group 3.7% (5/135).

Including the important covariates of the aforementioned generalized linear mixed model, there were no significant differences between the investigated treatment groups for the outcome variable CYC (*P* = 0.872; data not shown).

## Discussion

The aim of the present study was to evaluate the efficacy of an NSAID treatment in comparison to a reference therapy with solely local antibiotic treatment in the case of non-severe CM in cows with a long-lasting udder disease. In the case of CM, farmers assessed mastitis severity. Cows with severe cases were excluded from the trial and were treated systemically with antibiotics, according to the farms' treatment protocols. These animals were at risk of developing bacteremia, so irrespective of a previous onset of chronic mastitis, parenteral antibiotic treatment is recommended (33, 34).

The primary outcome in this trial was BC. Although BC risk of the reference group with antibiotic treatment was numerically higher with 48.1% compared to the NSAID group with 45.6%, no significant differences were confirmed by statistical analysis. As the CI spans the non-inferiority margin (–Δ), non-inferiority was inconclusive but very close to non-inferior for NSAID treatment in comparison to the solely antibiotic treatment (Figure 1). An inconclusive result could possibly occur due to a wide range of the CI. However, the confidence interval only slightly exceeded delta. With a larger number of cases, the non-inferiority could possibly be confirmed, as the span of the CI would become smaller. The non-inferiority margin of 15% was chosen according to previous CM trials (7, 29, 35, 36). Sample size was calculated to give the study sufficient power and to show a difference between test and reference therapy if there was a real difference of at least 15% according to Schukken et al. (7). The NSAID treatment in our study showed a numerically almost identical BC risk and no significant differences to the reference treatment; non-inferiority was inconclusive due to the lack of power. The overall BC risk was low in this study with 44.4%, as was the BC risk for CM cases treated with antibiotics (48.1%). This study exclusively included CM cases of cows with long-lasting udder diseases. Therefore, low likelihood of BC was expected, as studies had shown before (7, 12, 17, 37). In comparison, focusing on all occurring cases in a dairy herd, studies demonstrated BC risks of ~70% (7, 8, 18). The high differences in BC rates support the selection criteria used in this study to choose cows suffering from CM with a low likelihood of BC. Nevertheless, a tendency for the efficacy of antibiotic treatment against mastitis pathogens was shown.

A CC of the affected quarters was a secondary outcome in this study. Clinical cure risk was almost identical in both study groups (LSM; mixed model) and no statistically significant difference was found between the treatment groups. In the AB group, CC risk was slightly better with 0.5%. Again, due to the large calculated confidence interval of 17.6%, the statement regarding non-inferiority must also be inconclusive here. Other studies found a slightly higher likelihood of CC of ~60% for CM cases treated with antibiotics compared with our results, despite different, changing definitions of CC (7, 8). It is possible that CC risk worsens with increasing chronicity of mastitis.

The other secondary outcome variable was no R60. The probability of achieving no CM recurrence 60 d after the end of treatment was almost numerically identical for animals of the AB group (63.3%) and animals of the NSAID group (64.9%). Statistical analysis showed no significant differences between these two treatments. Non-inferiority was inconclusive because the CI also had a wide range (18.0%) and spanned –Δ and 0. Recurrences were observed only for clinically cured cases. Hence, the amount of evaluable cases was lower as in the models for the other outcomes and therefore CI increased. The no R60 risk of NSAID (64.9%) was numerically better than the rate of the AB treatment (63.3%). Comparing this result with those of previous trials which are also exclusively dealing with cows suffering from longer-lasting udder disease, no recurrence risks were similar (24). Studies performed without comparable selection criteria for enrolled CM cases described higher no recurrence rates of 80% within 60 d (13). CM is a disease with recurrent character (38). Cha et al. (39) showed that a cow with two CM cases in current lactation had a higher risk of contracting a third case. Thus, there was strong evidence that animals in this study were more likely to develop recurrent CM.

The evaluations of non-inferiority resulted in inconclusive findings for the targeted outcomes. A larger sample size of CM cases is required to confirm the detected results of the study and to make a clear statement on non-inferiority.

The specification of the non-inferiority margin is often controversial (40). As the control group received antibiotic treatment, primary outcome was BC. A non-inferiority margin of 0.15 was chosen because it had been used in antibiotic comparative studies and also in one of our studies when comparing antibiotic with an enzymatic mastitis therapy (7, 24, 25, 29, 36). Due to the very low BC rate to be expected when dealing with chronically udder-diseased cows, a wider margin might have been better suited for this trial. Based on the available literature on chronic mastitis, a large delta, as chosen in comparative studies with negative controls or placebo groups, did not seem appropriate to our study design. In addition, the choice of the primary outcome can be controversial, as BC is the actual goal of antibiotic mastitis therapy, but it is of little importance in the field. Ultimately, the acceptance in terms of cure rate reduction is a practical question.

The NSAID group received systemic treatment with ketoprofen (three applications at 24 h intervals with 3 mg of ketoprofen per kg bodyweight Kelaprofen®, Veyx-Pharma GmbH, Schwarzenborn, Germany). The prescribed withdrawal period on milk is 0 days, which ensures that there are no residues in the milk as a result of the usage. This is potentially the greatest advantage for farmers of this alternative treatment, as the milk can be sold again as soon as the cow is free of clinical signs of disease (although other NSAIDs might have a different prescribed withdrawal period on milk). This will also have a positive impact on farmers' costs due to the reduction in milk loss. Another advantage is that the risk from iatrogenic infection due to improper use of udder injectors is thus avoided. For cattle, according to the standard operating procedure of Kelaprofen®, the maximum treatment duration of 3 days should not be exceeded in order to avoid any unwanted side effects on the animals' gastrointestinal tract. The biggest challenge for farms might be the documentation of chronically udder-diseased cows and the implementation of the alternative treatment for these animals into existing treatment protocols and the daily procedure in cases of clinical mastitis. Avoiding useless antibiotic treatment complies with public demands and offers a sustainable treatment strategy in a broader perspective, but it can be challenging to convince farmers that these cows will not benefit from antibiotic treatment (5). The sharpened farmers' awareness of chronically udder-diseased cows in the herd might contribute to a targeted culling scheme and therefore might have a positive effect on the udder health at a herd level. Our intention was to reflect the situation in daily practice on dairy farms. Information about the causative pathogen was not available at the time of CM occurrence. Therefore, and because power calculations were made on overall therapy level, evaluations of treatment efficacy at a pathogen level gave no reliable results due to lack of power. Moreover, farmers were allowed to use their routine mastitis treatment procedure (AB) for CM cases of the reference group. That resulted in a wide range of used antibiotic products with different durations of treatment and withholding times. However, there were no indications of the various antimicrobial therapies influencing the study outcomes.

No completely untreated control group was included in our investigation. Mastitis is a painful condition for the cow. Therefore, for reasons of animal welfare a treatment is indicated. Also, so far there is no evidence-based information on the further course of CM in untreated animals. The participating farms were all economically oriented and the animals in the trial were in the regular production cycle. Thus, the formation of an untreated experimental group could not have been justified to the voluntarily participating farmers. Thus, we did not know whether the selection criteria chosen were correct to identify animals with a low probability of BC in lactation. As an additional outcome of the study, these selection criteria turned out to be well-adapted for this purpose. It is possible that stricter inclusion criteria (>3 clinical cases prior to the case under study and/or higher cow SCC cell count thresholds) would provide even clearer results.

Since the SCC significantly determines the value of the milk and thus influences the payment amount to the farmers, treatment is also intended to reduce the SCC of the affected quarter. In this study cows with mastitis history and persistent high cow SCC's were chosen and a low likelihood of BC was expected and proved. Antimicrobial treatment can solely target a BC and therefore a decreasing SCC can only be expected as a consequence of a reached BC (21). CYC rates in this study turned out quite low with 3.9% overall and with no significant differences between the treatment groups. Compared to a recently published study of Ziesch et al. (24), showing an overall CYC of 9.9%, this percentage is even lower. The authors suggested that a cow fulfilling the used selection criteria had a very low probability to recover from a physiological SCC in the affected udder quarter. In addition, the low CYC rate, accompanied by a CC rate of ~57%, was interpreted as an indication that the observed CM cases may turn subclinical with the remaining elevated quarter and therefore cow SCCs.

The actual very low BC rates achieved in this study demonstrate that an antibiotic treatment of cows with longer-lasting mastitis history can hardly be justified. Nevertheless, the milk of these cows is still saleable as long as they show no clinical symptoms. Therefore, farmers are particularly interested in a CC, a low recurrence rate, and a short time of discarding milk (41). With respect to the outcomes, the NSAID treatment seemed to achieve similar results in comparison to the reference group treated with antibiotics without having a withdrawal period for milk, which may decrease time of discarding milk, and a reduced risk of antibiotic residues. The results of this study will further encourage farmers and veterinarians to consider the impact of NSAID treatment, avoiding useless application of antibiotics in cases of chronically diseased cows.

## Conclusion

A randomized, multi-herd, non-inferiority study was conducted evaluating the efficacy of the test treatment ketoprofen in comparison to antibiotic treatment (AB; reference) of mild to moderate cases in cows with chronic mastitis. The test treatment showed no significant differences to the reference treatment with respect to the outcome variables BC, CC, no R60, and CYC. Solely NSAID therapy showed a numerically lower probability of BC and CC without significant differences to the reference treatment. NSAID treatment resulted in a numerically higher non-recurrence rate than the antibiotic treatment. The study findings indicate that solely using NSAID for treatment of mild to moderate CM in cows with long-lasting udder diseases may constitute an alternative therapy to reduce antibiotic usage. However, a greater sample size is needed to accomplish a reliable non-inferiority evaluation. Overall, the results for the different cure rates suggest that the used selection criteria of cows should be monitored in dairy herds. The quickest possible removal of such animals is recommended.

## Data Availability Statement

The raw data supporting the conclusions of this article will be made available by the authors, without undue reservation.

## Ethics Statement

The animal study was reviewed and approved by the animal welfare officer of the University of Veterinary Science Hannover; ethics committee and animal welfare officer of the University of Hanover.

## Author Contributions

VK: conceptualization and formal analysis. VK and EM-dV: methodology. VK and DK: software and data curation. VK and AS: validation and writer-original design preparation. EM-dV, VK, AS, NW, and YZ: investigation. VK, SL, DK, NW, and YZ: resources. VK, AS, DK, and SL: writer review and editing. AS: visualization. VK and SL: supervision and project management. All authors contributed to the article and approved the submitted version.

## Conflict of Interest

The authors declare that the research was conducted in the absence of any commercial or financial relationships that could be construed as a potential conflict of interest.

## References

[1] Falkenberg U Krömker V Heuwieser W Fischer-Tenhagen C. Survey on routines in udder health management and therapy of mastitis on German Dairy Farms. Milk Sci Int. (2019) 72:11–5. 10.25968/MSI.2019.2

[2] Oliveira L Ruegg PL. Treatments of clinical mastitis occurring in cows on 51 large dairy herds in Wisconsin. J Dairy Sci. (2014) 97:5426–36. 10.3168/jds.2013-7756 24997660

[3] Mansion-de Vries EM Lücking J Wente N Zinke C Hoedemaker M Krömker V. Comparison of an evidence-based and a conventional mastitis therapy concept with regard to cure rates and antibiotic usage. Milk Sci Int. (2016) 69:27–32. 10.25968/MSI.2016.6

[4] Kock J Wente N Zhang Y Paduch JH Leimbach S Klocke D. Udder health effects of an evidence-based mastitis therapy concept in Northwestern Germany. Milk Sci Int. (2018) 71:14–20. 10.25968/MSI.2018.4

[5] Schmenger A Leimbach S Wente N Zhang Y Biggs AM Krömker V. Implementation of a targeted mastitis therapy concept using an on-farm rapid test: antimicrobial consumption, cure rates and compliances. Vet Rec. (2020) 187:401. 10.1136/vr.105674 33024009

[6] Krömker V Paduch JH Klocke D Friedrich J Zinke C. Efficacy of extended intramammary therapy to treat moderate and severe clinical mastitis in lactating dairy cows. Berl Munch Tierarztl Wochenschr. (2010) 123:10–5. 20329647

[7] Schukken YH Zurakowski MJ Rauch BJ Gross B Tikofsky LL Welcome FL. Noninferiority trial comparing a first-generation cephalosporin with a third-generation cephalosporin in the treatment of nonsevere clinical mastitis in dairy cows. J Dairy Sci. (2013) 96:6763–74. 10.3168/jds.2013-6713 23958017

[8] Swinkels JM Krömker V Lam TJGM. Efficacy of standard vs. extended intramammary cefquinome treatment of clinical mastitis in cows with persistent high somatic cell counts. J Dairy Res. (2014) 81:424–33. 10.1017/S0022029914000442 25230074

[9] Ziesch M Krömker V. Factors influencing bacteriological cure after antibiotic therapy of clinical mastitis. Milk Sci Int. (2016) 69:7–14. 10.25968/MSI.2016.2

[10] Trevisi E Zecconi A Cogrossi S Razzuoli E Grossi P Amadori M. Strategies for reduced antibiotic usage in dairy cattle farms. Res Vet Sci. (2014) 96:229–33. 10.1016/j.rvsc.2014.01.001 24508188

[11] Pinzón-Sánchez C Ruegg PL. Risk factors associated with short-term post-treatment outcomes of clinical mastitis. J Dairy Sci. (2011) 94:3397–410. 10.3168/jds.2010-3925 21700025

[12] Sol J Sampimon OC Barkema HW Schukken YH. Factors associated with cure after therapy of clinical mastitis caused by *Staphylococcus aureus*. J Dairy Sci. (2000) 83:278–84. 10.3168/jds.S0022-0302(00)74875-2 10714861

[13] Bradley AJ Green MJ. Factors affecting cure when treating bovine clinical mastitis with cephalosporin-based intramammary preparations. J Dairy Sci. (2009) 92:1941–53. 10.3168/jds.2008-1497 19389951 PMC2675183

[14] Swinkels JM Cox P Schukken YH Lam TJGM. Efficacy of extended cefquinome treatment of clinical *Staphylococcus aureus* mastitis. J Dairy Sci. (2013) 96:4983–92. 10.3168/jds.2012-6197 23706485

[15] GVA. Guidelines for Combating Bovine Mastitis as a Stock Problem. 5th ed. Gießen: German Veterinary Association (2012).

[16] Grieger AS Zoche-Golob V Paduch JH Hoedemaker M Krömker V. Recurrent clinical mastitis in dairy cattle – importance and causes. Tierarztl Prax Ausg G. (2014) 42:156–62. 10.1055/s-0038-1623218 24920089

[17] Linder M Paduch JH Grieger AS Mansion-de Vries E Knorr N Zinke C. Cure rates of chronic subclinical *Staphylococcus aureus* mastitis in lactating dairy cows after antibiotic therapy. Berl Munch Tierarztl Wochenschr. (2013) 126:291–6. 23901584

[18] Schmenger A Krömker V. Characterization, cure rates and associated risks of clinical mastitis in Northern Germany. Vet Sci. (2020) 7:170. 10.3390/vetsci7040170 33153084 PMC7712256

[19] Wente N Grieger AS Klocke D Paduch JH Zhang Y Leimbach S. Recurrent mastitis–persistent or new infections? Vet Microbiol. (2020) 244:108682. 10.1016/j.vetmic.2020.108682 32402348

[20] Krömker V Friedrich J. Recommendations for diagnostic measures regarding mastitis control on herd level. Prakt Tierarzt. (2011) 92:516–24.

[21] Degen S Paduch JH Hoedemaker M Krömker V. Factors affecting the probability of bacteriological cure of bovine mastitis. Tierarztl Prax Ausg G. (2015) 43:222–7. 10.15653/TPG-141082 25960107

[22] Krömker V Paduch JH Abograra I Zinke C Friedrich J. Effects of an additional nonsteroidal anti-inflammatory therapy with carprofen (Rimadyl Rind®) in cases of severe mastitis of high yielding cows. Berl Munch Tierarztl Wochenschr. (2011) 124:161−7. 21465772

[23] McDougall S Bryan MA Tiddy RM. Effect of treatment with the nonsteroidal antiinflammatory meloxicam on milk production, somatic cell count, probability of re-treatment, and culling of dairy cows with mild clinical mastitis. J Dairy Sci. (2009) 92:4421–31. 10.3168/jds.2009-2284 19700702

[24] Ziesch M Wente N Zhang Y Zaremba W Engl S Krömker V. Noninferiority trial investigating the efficacy of a nonantibiotic intramammary therapy in the treatment of mild-to-moderate clinical mastitis in dairy cows with longer lasting udder diseases. J Vet Pharmacol Ther. (2018) 41:11–21. 10.1111/jvp.12415 28449183

[25] Piaggio G Elbourne DR Altman DG Pocock SJ Evans SJW. Reporting of non-inferiority and equivalence randomized trials: an extension of the CONSORT statement. JAMA. (2009) 295:1152–60; 1842. 10.1001/jama.295.10.1152 16522836

[26] O‘Connor AM Sargeant JM Gardner IA Dickson JS Torrence ME Dewey CE. The REFLECT statement: methods and processes of creating reporting guidelines for randomized controlled trials for livestock and food safety. J Vet Intern Med. (2010) 24:57–64. 10.1111/j.1939-1676.2009.0441.x 20002546

[27] EMEA. VICH Topic GL9 (GCP): Guideline on Good Clinical Practices. London: The European Agency for the Evaluation of Medicinal Products (2000).

[28] Schukken YH Bennett GJ Zurakowski MJ Sharkey HL Rauch BJ Thomas MJ. Randomized clinical trial to evaluate the efficacy of a 5-day ceftiofur hydrochloride intramammary treatment on nonsevere gram-negative clinical mastitis. J Dairy Sci. (2011) 94:6203–15. 10.3168/jds.2011-4290 22118109

[29] Vasquez AK Nydam DV Capel MB Ceglowski B Rauch BJ Thomas MJ. Randomized noninferiority trial comparing 2 commercial intramammary antibiotics for the treatment of nonsevere clinical mastitis in dairy cows. J Dairy Sci. (2016) 99:8267–81. 10.3168/jds.2016-11258 27522408

[30] GVA Guidelines for Aseptic Milk Sampling and Guidelines to Isolate and Identify Mastitis Pathogens. 2nd ed. Gießen: German Veterinary Association (2009).

[31] IDF. Suggested Interpretation of Mastitis Terminology. Bulletin of the IDF 338. (1999). Brussels: International Dairy Federation (1999).

[32] Watts JL Salmon SA Yancey RJJ. Use of modified Rambach agar to differentiate *Streptococcus uberis* from other mastitis streptococci. J Dairy Sci. (1993) 76:1740–3. 10.3168/jds.S0022-0302(93)77506-2 8326035

[33] Wenz JR Barrington GM Garry FB McSweeney KD Dinsmore RP Goodell G. Bacteremia associated with naturally occurring acute coliform mastitis in dairy cows. J Am Vet Med Assoc. (2001) 219:976–81. 10.2460/javma.2001.219.976 11601796

[34] Erskine RJ Wagner S DeGraves FJ. Mastitis therapy and pharmacology. Vet Clin North Am Food Anim Pract. (2003) 19:109–38. 10.1016/S0749-0720(02)00067-1 12682938

[35] Schukken YH Deluyker HA. Design of field trials for the evaluation of antibacterial products for therapy of bovine clinical mastitis. J Vet Pharmacol Ther. (1995) 18:274–83. 10.1111/j.1365-2885.1995.tb00591.x 8583540

[36] Deluyker HA Chester ST van Oye SN. A multilocation clinical trial in lactating dairy cows affected with clinical mastitis to compare the efficacy of treatment with intramammary infusions of a lincomycin/neomycin combination with an ampicillin/cloxacillin combination. J Vet Pharmacol Ther. (1999) 22:274–82. 10.1046/j.1365-2885.1999.00205.x 10499240

[37] Krömker V Wente N Zhang Y Bolte J Renner R Schmenger A. Comparison of a non-antibiotic treatment with an antibiotic treatment of chronic mastitis. Milk Sci Int. (2019) 72:34−8. 10.25968/MSI.2019.631288901

[38] Schukken YH Bar D Hertl J Gröhn YT. Correlated time to event data: modeling repeated clinical mastitis data from dairy cattle in New York State. Prev Vet Med. (2010) 97:150–6. 10.1016/j.prevetmed.2010.09.012 21035216

[39] Cha E Hertl J Schukken Y Tauer L Welcome F Gröhn Y. Evidence of no protection for a recurrent case of pathogen specific clinical mastitis from a previous case. J Dairy Res. (2016) 83:72–80. 10.1017/S002202991500062X 26568557

[40] Powers JH Fleming TR. Non-inferiority trials: clinical understandings and misunderstandings. Clin Invest. (2013) 3:215–8. 10.4155/cli.12.157 24563733 PMC3929272

[41] Ruegg PL. The Application of Evidence Based Veterinary Medicine to Mastitis Therapy. Santiago: World Buiatrics Congress (2010).

